# Clinical Significance of Soluble L1CAM Serum Levels in Patients with High-Risk Endometrial Cancer

**DOI:** 10.3390/biomedicines13112670

**Published:** 2025-10-30

**Authors:** Antonella Ravaggi, Cosetta Bergamaschi, Laura Zanotti, Elisa Gozzini, Marina Momi, Germana Tognon, Franco Odicino, Eliana Bignotti

**Affiliations:** 1 Department of Obstetrics and Gynecology, ASST Spedali Civili di Brescia, 25123 Brescia, Italy; c.bergamaschi001@studenti.unibs.it (C.B.); laura.zanotti@asst-spedalicivili.it (L.Z.); elisa.gozzini@asst-spedalicivili.it (E.G.); germana.tognon@asst-spedalicivili.it (G.T.); franco.odicino@unibs.it (F.O.); 2 Department of Clinical and Experimental Sciences, University of Brescia, 25123 Brescia, Italy; marinamomi@hotmail.it; 3 Angelo Nocivelli Institute for Molecular Medicine Research Center, ASST Spedali Civili di Brescia, University of Brescia, 25123 Brescia, Italy; 4 Residency Program for Clinical Pathology and Clinical Biochemistry, University of Brescia, 25123 Brescia, Italy

**Keywords:** endometrial cancer, L1CAM, serum, prognosis

## Abstract

**Background/Objectives**: Despite advances in targeted therapies, a substantial proportion of high-risk endometrial carcinomas (EC) do not respond to treatment and have a poor prognosis. The identification of prognostic and predictive biomarkers to improve patient stratification is therefore a clinical priority. L1 cell adhesion molecule (L1CAM) is a promising biomarker in EC; however, its soluble circulating form (sL1CAM) has been poorly investigated. This study aimed to evaluate the prognostic and predictive significance of sL1CAM in high-risk ECs. **Methods**: High-risk EC patients, treated with surgery and platinum-based adjuvant chemotherapy, were retrospectively enrolled. sL1CAM levels were quantified in 72 preoperative serum samples by enzyme-linked immunosorbent assay (ELISA). **Results**: High sL1CAM levels were associated with advanced age and non-endometrioid histology. Across the entire patient cohort, higher sL1CAM concentrations significantly correlated with worse prognosis in terms of DSS and PFS in univariate (DSS: HR = 2.22, *p* = 0.028; PFS: HR = 1.21, *p* = 0.041) and multivariate (DSS: HR = 2.13, *p* = 0.041; PFS: HR = 1.93, *p* = 0.048) analyses. Stratification by histological type revealed a significant prognostic association only in the endometrioid subgroup, both in univariate and multivariate analyses. Moreover, in this subgroup, elevated sL1CAM levels were associated with shorter time to recurrence after chemotherapy, both in univariate (PFI: HR = 2.69, *p* = 0.027) and multivariate (PFI: HR = 2.97, *p* = 0.017) analysis, and significantly predicted relapse within 6 months (OR = 7.83, *p* = 0.027). **Conclusions**: sL1CAM is associated with poor prognosis in high-risk EC and seems to be associated with platinum response in endometrioid tumors. These findings support its potential role as a biomarker to improve risk stratification, warranting validation in larger, prospective studies.

## 1. Introduction

Endometrial cancer (EC) is the most frequent gynecological cancer in developed countries and the sixth most common cancer among women worldwide [1,2]. EC shows an increasing incidence rate, due to population aging, and the rising prevalence of obesity. Although most neoplasms are diagnosed at an early stage and the 5-year prognosis exceeds 80%, some histological types (especially non-endometrioid) present at an advanced stage, with an overall survival rate of 22% (https://seer.cancer.gov/statfacts/html/corp.html, accessed on 13 August 2025). Collectively, these factors have made EC an increasingly important public health challenge [2,3].

When EC is diagnosed at an early stage [1,2], surgical management, followed by adjuvant therapy when clinically indicated, is generally associated with a favorable prognosis. However, approximately 15–20% of patients present with high-risk disease, characterized by advanced FIGO stage, high-grade tumor, substantial lymphovascular space invasion (LVSI), and non-endometrioid histological type [4,5]. High-risk ECs are associated with an increased risk of recurrence and, despite advancements in therapeutic modalities, curative treatment options for many of these patients remain limited, with a generally poor prognosis [1,5,6].

Recently, a new molecular classification of EC has been defined by The Cancer Genome Atlas (TCGA). According to this prognostic classification, which is based on the tumor’s genomic profile, EC can be divided into four groups with distinct progression-free survival (PFS): polymerase-E (POLE) mutated tumors, characterized by excellent survival; mismatch repair deficient (MMRd) tumors and EC with non-specific mutation profile (NSMP), both showing intermediate survival; and p53 mutated tumors, associated with poor prognosis [7]. Despite the growing adoption of this classification system, the identification of novel prognostic biomarkers remains crucial for effective patient risk stratification, guiding personalized therapeutic strategies, and accurately predicting disease progression [6].

L1CAM (L1-cell adhesion molecule) is a transmembrane glycoprotein of the immunoglobulin superfamily, closely related to neural adhesion molecules and known for its role in nerve cell function [8,9]. L1CAM is also overexpressed in various types of human cancer, including gynecological tumors, such as ovarian cancer and EC [8,9,10,11], linked to poor prognosis, aggressive and invasive phenotype, advanced disease stages, metastasis, and resistance to chemotherapy [8,9,10].

In cancer cells, L1CAM ectodomain is not only expressed on the cell surface, but can also be shed into the extracellular environment as a soluble form of ~200 kDa, through proteolytic cleavage mediated by two A Disintegrin and Metalloprotease (ADAM) enzymes, specifically ADAM10 and ADAM17, or matrix metalloprotease 16 (MMP-16) [12,13,14].

In the literature, numerous studies have investigated the expression of L1CAM in EC tissue using immunohistochemistry [15,16,17,18,19,20,21]. Although soluble L1CAM (sL1CAM) has been detected in the serum and ascites of patients with epithelial tumors, such as EC and ovarian carcinoma [22], research assessing circulating protein levels remains limited [23,24,25,26,27].

The aim of our study was to evaluate preoperative serum levels of sL1CAM in patients with high-risk EC, in order to investigate a possible correlation between this biomarker and the patients’ clinicopathological characteristics. Furthermore, we analyzed whether sL1CAM levels at the time of diagnosis could have prognostic value and predict response to platinum-based adjuvant chemotherapy.

## 2. Materials and Methods

### 2.1. Patients and Biological Samples

Our retrospective study was performed on a total of 72 patients with EC diagnosed and treated at the Division of Obstetrics and Gynecology of ‘ASST Spedali Civili di Brescia’ (Brescia, Italy), between March 2004 and December 2020. The inclusion criteria were: (i) histologically confirmed EC (endometrioid, serous, mixed, and clear cell histological types), (ii) high–intermediate, high or advanced metastatic risk-group according to ESGO/ESTRO/ESP 2021 guidelines [7], (iii) administration of platinum-based adjuvant chemotherapy, and (iiii) availability of at least one frozen serum sample collected prior to any treatment (surgery or neo-adjuvant chemotherapy). Patients with synchronous cancer or with a history of malignancy in the 5 years prior to the EC diagnosis were excluded from the study. Clinical and follow-up data were acquired from the original reports.

The stage of disease was assessed according to the International Federation of Gynecology and Obstetrics (FIGO) staging system revised in 2009. Risk group stratification was performed according to the ESGO/ESTRO/ESP guidelines (molecular classification unknown) [7]. The characteristics of the patients are summarized in Table 1.

All patients received adjuvant therapy: 35 (48.6%) were treated exclusively with platinum-based chemotherapy and 37 (51.4%) with chemotherapy plus radiotherapy.

Follow-up data were updated until May 2025 for patients who were still alive, and this date was used as the censoring point in survival analyses (median follow-up, 95 months, range 27–232 months). At the time of the last follow-up, 27 patients (37.5%) were alive without evidence of disease, 10 (13.9%) were alive with disease, 33 (45.8%) were dead from disease, and 2 (2.8%) died from other causes.

The study was conducted in accordance with the principles outlined in the Declaration of Helsinki and received approval from the Research Review Board–Ethics Committee of Brescia, Italy (protocol NP3784, approval: 2021-08-24). All participants provided written informed consent.

### 2.2. Measurement of sL1CAM in Serum

All blood samples were drawn from patients in a fasting state and strictly before surgery or neo-adjuvant chemotherapy. Serum was separated by centrifugation at 1500× *g* for 10 min within 1 h of collection, frozen in liquid nitrogen and subsequently stored at −80 °C in the biorepository of the “A. Nocivelli Institute of Molecular Medicine” until analysis. Serum levels of sL1CAM were analyzed using an enzyme-linked immunosorbent assay (L1CAM ELISA kit, catalog number MBS2023094-96, MyBioSource, San Diego, CA, USA), following the manufacturer’s instructions. Briefly, before performing the immunoassay, serum samples were thawed and diluted 1:5 in Phosphate-Buffered Saline (PBS). One hundred microliters of standard and diluted serum samples were analyzed in duplicate, and plates were read at 450 nm on a SpectraMax ABS Plus-R plate reader (Molecular Devices, LLC., San Jose, CA, USA). According to the manufacturer, the dynamic detection range of the assay was from 15.6 to 1000 pg/mL, with intra-assay and inter-assay coefficients of variation (CV) of <10% and <12%, respectively. The samples resulted above the highest value of the standard curve were repeated at a higher dilution.

### 2.3. Statistical Methods

For the association with clinical characteristics and the survival analysis, sL1CAM levels were transformed using the natural logarithm and subsequently dichotomized into high and low expression groups with the median as a cutoff. In addition, to explore a more outcome-oriented stratification, we applied the Maximally Selected Rank Statistics method [28] to determine the optimal cut-off for predicting survival. Associations with clinical characteristics were assessed using the chi-square test. Survival analysis was performed using Cox proportional hazards regression and Kaplan–Meier survival curves. For survival analysis, three endpoints were evaluated: Disease-Specific Survival (DSS), Progression-Free Survival (PFS), and Platinum-Free Interval (PFI). DSS was defined as the time from the date of diagnosis to the date of cancer-related death or last follow-up. PFS was defined as the time from diagnosis to disease progression, recurrence, or death. PFI was calculated from the end of adjuvant platinum-based chemotherapy to the date of recurrence or progression. For all endpoints, patients without an event were censored at the date of last follow-up. A *p*-value < 0.05 was considered statistically significant. All statistical analyses were performed using Jamovi 2.6.44.0 (Jamovi Project, Sydney, Australia).

## 3. Results

### 3.1. High sL1CAM Levels Are Associated with Advanced Age and Non-Endometrioid Histological Type

Serum concentrations of sL1CAM were measurable in all samples, with a median value of 3140.9 pg/mL (range: 1777.3–7972.7). This median was used as a cut-off to dichotomize patients into high and low expression groups. To validate the robustness of this stratification, we also applied the Maximally Selected Rank Statistics method, which identified an optimal cut-off of 3133.8 pg/mL for predicting disease-specific survival and it classified patients identically to the median-based cut-off. High sL1CAM levels were significantly associated with older age (*p* = 0.005) and non-endometrioid histological type (*p* < 0.001). L1CAM levels tended to be higher in the high-risk and advanced-metastatic groups compared to the high–intermediate group; however, this difference did not reach statistical significance (*p* = 0.061). No significant associations were observed between sL1CAM levels and other clinical characteristics, including FIGO stage, tumor grade, depth of myometrial invasion, lymph node metastasis and lymph vascular space invasion (see Table 1).

### 3.2. High sL1CAM Levels Predict Poor Outcome in Endometrioid EC and p53-wt EC

Higher concentrations of sL1CAM were significantly correlated with worse prognosis both in terms of DSS (HR = 2.22, *p* = 0.028) and PFS (HR = 1.91, *p* = 0.041) in univariate analysis. As expected, older age, non-endometrioid histological type, advanced FIGO stage, and the presence of lymph node metastases were also associated with poorer outcomes (Table 2).

In multivariate analysis, sL1CAM confirmed its statistical significance regardless of FIGO stage and tumor grade for both DSS (HR = 2.13, *p* = 0.041) and PFS (HR = 1.93, *p* = 0.048).

**Figure 1:**
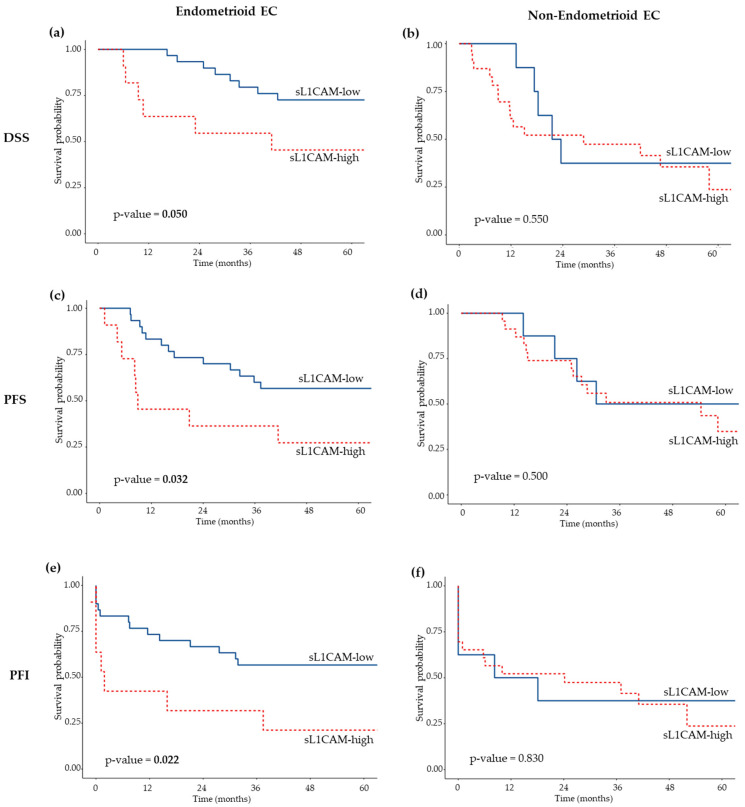
Kaplan–Meier survival curves showing DSS (**a**,**b**), PFS (**c**,**d**), and PFI (**e**,**f**) according to sL1CAM levels in EC patients with endometrioid (**a**,**c**,**e**) and non-endometrioid (**b**,**d**,**f**) histological types. Log-rank *p*-values are indicated in each panel.

Considering the correlation between L1CAM levels and histological type, we evaluated its prognostic value separately in patients with endometrioid (EEC) and non-endometrioid (non-EEC) EC. sL1CAM levels were significantly associated with both DSS and PFS only in the endometrioid cohort, while no significant association was observed in patients with non-EEC (see Figure 1, panels a–d, for Kaplan–Meier survival curves). In the EEC cohort, patients with high level of sL1CAM had a significantly worse prognosis with a HR of 2.90 (*p* = 0.045) for DSS and a HR of 2.58 (*p* = 0.033) for PFS, compared to those with low sL1CAM levels in univariate analysis; the 5-year DSS was 45.5% (95% CI: 23.8–86.8) and 72.6% (95% CI, 58.1–90.7) for L1CAM-high and L1CAM-low patients, respectively, while 5 years after diagnosis, 27.3% (95% CI: 10.4–71.6) of L1CAM-high patients and 56.7% (95% CI, 41.4–77.5) of L1CAM-low patients had not experienced disease recurrence. After adjusting for stage and grade in the multivariate analysis, the difference between L1CAM-high and L1CAM-low EEC groups remained significant for both DSS (HR = 2.96, *p* = 0.043) and PFS (HR = 2.45, *p* = 0.045) (Table 3). The prognosis of sL1CAM-high EEC did not differ from that of non-EEC for both DSS (HR = 1.03, 95% CI: 0.41–2.61, *p* = 0.948) and PFS (HR = 1.41, 95% CI: 0.62–3.21, *p* = 0.411). This similarity persisted in multivariate analysis after adjustment for FIGO stage and tumor grade: DSS (HR = 0.94, 95% CI: 0.36–2.42, *p* = 0.891) and PFS (HR = 1.19, 95% CI: 0.52–2.74, *p* = 0.683).

**Figure 2:**
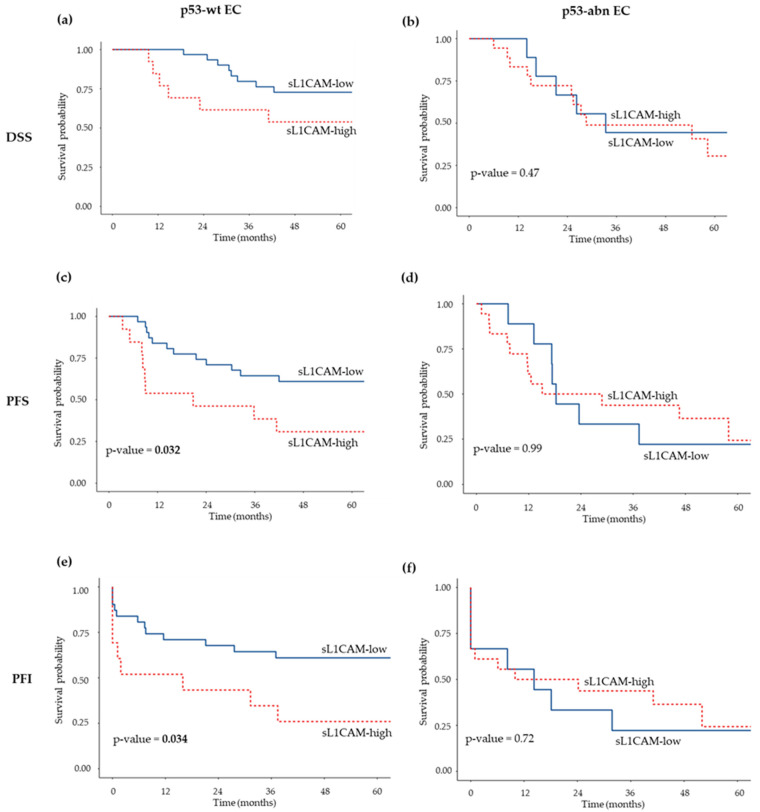
Kaplan–Meier survival curves showing DSS (**a**,**b**), PFS (**c**,**d**), and PFI (**e**,**f**) according to sL1CAM levels in patients with p53-wt EC (**a**,**c**,**e**) and p53-abn EC (**b**,**d**,**f**). Log-rank *p*-values are indicated in each panel.

A total of 27 out of 72 EC cases were classified as p53-abnormal (p53-abn) based on immunohistochemical analysis. We observed a significant direct association between p53-abn status and higher sL1CAM levels, as 66% of p53-abn patients belonged to the sL1CAM-high group compared with 41% of those with p53-wt tumors (*p* = 0.035). Therefore, we subsequently analyzed the prognostic significance of sL1CAM separately in p53-wt and p53-abn patients. In the p53-wt group, high sL1CAM levels were significantly associated with shorter PFS in univariate (HR = 2.48, 95% CI: 1.05–5.82, *p* = 0.038) and showed a borderline association in multivariate analysis (HR = 2.34, 95% CI: 0.98–5.59, *p* = 0.055). No significant association was observed for DSS either in univariate (HR = 2.19, 95% CI: 0.77–6.25, *p* = 0.141) or multivariate analysis (HR = 2.17, 95% CI: 0.75–6.25, *p* = 0.153), after adjustment for FIGO stage and tumor grade (see Figure 2, panels a,c, for Kaplan–Meier survival curves). In the p53-abn group, sL1CAM did not appear to influence prognosis (see Figure 2, panels b,d, for Kaplan–Meier survival curves).

### 3.3. Elevated sL1CAM Levels Are Associated with Shorter Time to Recurrence and Predict Early Relapse After Platinum-Based First-Line Chemotherapy

We further assessed whether sL1CAM status could help in predicting the timing of recurrence after platinum-based adjuvant chemotherapy, beyond its role as a prognostic factor. As shown in Table 2, patients with high sL1CAM were characterized by a trend toward shorter PFI (HR = 1.82, *p* = 0.059).

Subsequently, we stratified patients by histological type. As observed for DSS and PFS, high sL1CAM levels were associated with a shorter PFI exclusively in the EEC subgroup, both in univariate (HR = 2.69, *p* = 0.027) and multivariate (HR = 2.97, *p* = 0.017) analyses (Table 3); the five-years PFI was 21.2% (95% CI: 6.3–71.6) and 56.7% (95% CI: 41.4–77.5) for L1CAM-high and L1CAM-low patients, respectively. No significant association was observed among the non-EEC subgroup (Figure 1, panel f).

To explore differences in recurrence patterns after platinum-based chemotherapy, patients were classified as ‘early-relapsing’ (PFI < 6 months) or ‘late-relapsing’ (PFI > 12 months). In the EEC cohort, 64% of sL1CAM-high patients were early-relapsing, compared to 23% of sL1CAM-low patients. This difference in time to recurrence was statistically significant (Chi-square test, *p* = 0.016).

**Figure 3:**
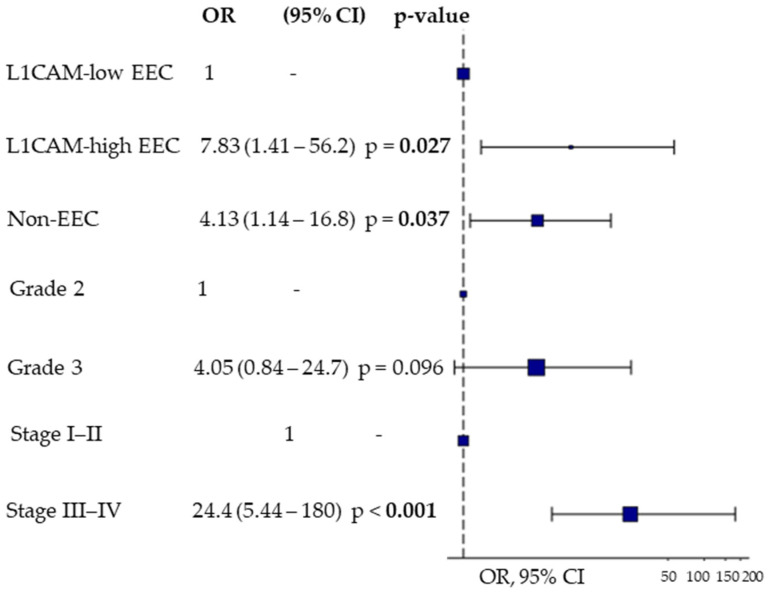
Multivariate Odds Ratio Plot for predicting early (PFI < 6 months) vs. late relapse (PFI > 12 months) after platinum-based first-line chemotherapy using sL1CAM levels, tumor grade, and FIGO stage. Endometrioid tumors (EEC) were stratified according to sL1CAM levels, while non-endometrioid tumors (non-EEC) were considered as a single group.

Thereafter, we assessed the ability of sL1CAM to predict early versus late relapse following platinum-based chemotherapy using logistic regression analysis. In the univariate analysis, taking EEC patients with low sL1CAM levels as the reference group, those with high sL1CAM levels had an odds ratio (OR) of 5.75 (95% CI: 1.35–28.1, *p* = 0.022) for experiencing relapse within 6 months, while non-EEC patients had an OR of 3.08 (95% CI: 1.05–9.71, *p* = 0.045). This association remained statistically significant in multivariate analysis after adjusting for stage and grade (Figure 3).

Finally, we stratified patients according to p53 status and found that, only in the p53-wt subgroup, high sL1CAM levels were significantly associated with worse PFI in both univariate (HR = 2.50, 95% CI: 1.06–5.90, *p* = 0.037) and multivariate analysis (HR = 2.46, 95% CI:1.03–5.86, *p* = 0.042), after adjustment for stage and grade. (Figure 2, panels e–f).

## 4. Discussion

The current treatment of high-risk EC is primarily based on surgery and adjuvant therapy. However, despite the significant improvement in patient prognosis with the introduction of innovative approaches, such as immunotherapy and PARP inhibitors, a substantial proportion of patients still experience poor survival [6]. Therefore, there is an increasing interest in identifying novel prognostic biomarkers to enable accurate preoperative stratification and guide subsequent therapeutic approaches, ultimately allowing for optimal and personalized patient management. L1CAM has emerged as a promising prognostic biomarker and its expression has been well characterized at the tissue level using immunohistochemistry or RT-qPCR in various tumors including EC [29]. Specifically, overexpression of L1CAM in EC tissues has been demonstrated to correlate with unfavorable clinical outcomes and reduced response to platinum-based chemotherapy in EC [15,26,30,31]. Confirming these findings through liquid biopsy would offer several advantages: non-invasive sampling, information about risk stratification prior to surgery, the possibility of repeated testing during treatment and follow-up and the ability to collect samples at recurrence even in the absence of relapsed tissue. Only a few studies in the literature have investigated circulating sL1CAM levels in patients with EC [23,25,26].

In this context, our study was the first to measure L1CAM serum concentration in a cohort of high-risk EC patients prior to surgery. Firstly, we analyzed the correlation between sL1CAM levels and the patients’ clinicopathological features. High sL1CAM levels were significantly associated with older age and non-endometrioid histological type. Our results are in agreement with those obtained by Tangen et al. [26], who investigated sL1CAM levels in plasma samples of 372 EC patients, whereas Sertel et al. [25] and Bednarikova et al. [23] reported significant association of sL1CAM only with histotype and age, respectively. In contrast to our findings, Tangen et al. and Sertel et al. [25,26] also observed association between elevated sL1CAM levels and advanced stage, deep myometrial invasion (M2), higher histological grade, and substantial LVSI. These discrepancies may be explained by differences in study populations: while all our patients were high-risk ECs, those in previous studies included consecutively enrolled patients more frequently presenting with early-stage and low-grade tumors. Then, we evaluated the association between sL1CAM and prognosis. Our results demonstrate that sL1CAM is a negative prognostic factor for both DSS and PFS, regardless of stage and grade, in the entire cohort of high-risk EC patients, and particularly in the endometrioid subgroup. These findings are consistent with Tangen et al., who reported that high levels of sL1CAM were significantly associated with worse DSS in univariate analysis in both the entire patient population and the low-risk histology subgroup at curettage, represented by G1/G2 endometrioid EC [26]. Unlike our study, they did not observe significance in multivariate analysis, likely due again to the different patient characteristics. To our knowledge, Tangen’s study is the only one to have evaluated the prognostic significance of circulating sL1CAM in the blood of EC patients. Instead, previous investigations, including one recently published by our group, have described an association between high L1CAM expression, assessed at the tissue level by immunohistochemistry and worse prognosis in EC patients [15,32,33]. Notably, the prognostic relevance of L1CAM restricted to endometrioid EC, but not observed in non-endometrioid tumors, had already been reported in a collaborative ENITEC study evaluating its tissue expression in a series of 1199 EC cases [33]. Notably, in our study, the prognosis of sL1CAM-high endometrioid EC was comparable to that of non-endometrioid tumors. This suggests that circulating sL1CAM may help identify a biologically aggressive subgroup within endometrioid EC, irrespective of FIGO stage and tumor grade. A possible explanation for the finding that sL1CAM is prognostic only in endometrioid EC could be the heterogeneity of this histological subtype, which may include all four molecular groups, whereas non-endometrioid ECs represent a more homogeneous group characterized by an inherently poor prognosis. In line with this, tissue-based studies have shown greater variability of L1CAM expression within endometrioid ECs, whereas non-endometrioid ECs more consistently display high L1CAM expression [33,34]. In our cohort 12% of endometrioid ECs and 71% of non-endometrioid ECs were p53-abnormal and we observed that the prognostic value of sL1CAM was limited to patients with p53-wt EC. This biological context may explain why sL1CAM adds prognostic information in endometrioid, but not in non-endometrioid ECs. Moreover, TP53 mutations are frequently the driver events in non-endometrioid ECs, and p53 mutation is recognized as a strong independent prognostic factor, more powerful than histological characteristics alone, identifying the subgroup of ECs with the worst overall prognosis [35].

The biological basis of the role of L1CAM has not yet been adequately explored in EC. Many studies on cancer cell lines have demonstrated the role of full-length L1CAM in promoting tumor progression. Specifically, L1CAM has been shown to stimulate cell proliferation, migration and invasion in gastric cancer [36], non-small-cell lung cancer [37], and pancreatic cancer [38] to protect cells from apoptosis and to confer a phenotype resistant to drugs in ovarian cancer [39] and retinoblastoma [13]; and to promote epithelial to mesenchymal transition and formation of cancer initiating cells in EC [40].

The biological role of extracellular sL1CAM has been less extensively studied compared to the full-length form of L1CAM, but there is evidence suggesting its role in promoting tumor progression [14]. Specifically, sL1CAM can bind to L1CAM on the same cell or nearby cells, as well as to integrins and tyrosine kinase receptors (RTKs), which activates ERK1/2 and prevents cell death [41]. Moreover, in ovarian cancer cell lines sL1CAM from ascitic fluid can bind to integrins at the cell surface and induce cell migration in a dose-dependent manner, that is partially blocked by incubation with antibodies anti-integrins α5β1, αvβ3, and αvβ5 [42]. Notably, αv integrins are implicated in the activation of Ras, Raf-1, and ERK signaling pathways during the process of angiogenesis [43].

In conclusion, in the present study, we showed that high sL1CAM levels were associated with shorter PFI in EEC and may serve as a predictor of early recurrence after platinum-based chemotherapy. Consistently, our group recently demonstrated that blocking L1CAM in primary EC cell lines significantly enhanced their sensitivity to carboplatin, suggesting a functional relationship between L1CAM expression and the response of EC cells to platinum-based chemotherapy [15]. To date, no direct link between sL1CAM and platinum resistance in EC has been demonstrated in in vitro experiments. However, Soeck et al. reported that sL1CAM can protect cells from apoptosis in ovarian carcinoma cell lines [39]. Additionally, the authors reported that the anti-apoptotic mechanism associated with circulating L1CAM, in the human epithelial kidney cell line HEK293, appears to be mediated by the activation of ERKα, FAK, and by an increased expression of the anti-apoptotic protein Bcl-2. A further study [44] suggested that L1CAM may contribute to cisplatin resistance in epithelial ovarian cancer (EOC). The authors demonstrated that overexpression of the transcription factor TWIST1, a known regulator of epithelial–mesenchymal transition (EMT), promotes a platinum-resistant phenotype through activation of the PI3K/Akt pathway. Specifically, increased TWIST1 expression in EOC cells was shown to enhance cisplatin resistance, partly via upregulation of L1CAM. Moreover, silencing of L1CAM in cells with high TWIST1 levels restored drug sensitivity, thus supporting a direct role of L1CAM in promoting chemoresistance. Although these findings relate to the membrane-bound form of L1CAM, it is plausible to hypothesize that sL1CAM, through paracrine or autocrine signaling mechanisms, could modulate cell survival and contribute to chemotherapy resistance. However, since no studies to date have directly linked the functional role of sL1CAM to platinum resistance in tumors, further research is necessary to clarify this potential association. Nonetheless, these preliminary insights suggest that sL1CAM might serve as a promising biomarker of platinum response, a hypothesis that warrants confirmation in prospective randomized clinical studies. Integrating sL1CAM quantification into the therapeutic decision-making process, alongside molecular classification, could help guide patients who may not benefit from platinum-based chemotherapy toward alternative treatment regimens, such as immune checkpoint inhibitors for POLE-mutated or MMRd EC, and hormonal therapies or PI3K-AKT-mTOR inhibitors for NSMP EC [15].

The main limitation of our study is the lack of EC molecular characterization, as this was not yet part of clinical practice when most patients were enrolled. Currently, molecular classification has been incorporated into the FIGO staging system, and the detection of p53-abnormal or POLE-mutated status in stage I or II leads to upstaging or downstaging, respectively [45]. Without molecular stratification, we cannot exclude that the prognostic significance of sL1CAM may have a different impact among molecular subgroups. Our future plans include conducting a multicenter prospective study to determine whether sL1CAM levels remain an independent prognostic and predictive factor within molecular subgroups. This investigation could have a particularly significant impact in the NSMP group, which is a clinically heterogeneous category lacking clear prognostic or predictive biomarkers where sL1CAM assessment could provide substantial added value to help guide more targeted therapy. Therefore, our present findings should be considered hypothesis-generating, and their clinical applicability will need to be validated in molecularly stratified cohorts to align with the current standard of care.

A second key aim of this future study, achievable only with a large patient cohort, will be to establish an optimal cut-off in an initial discovery set and validate it in an independent cohort for a future use of sL1CAM as a biomarker in the clinical setting. Evaluation of sL1CAM levels could represent a minimally invasive approach to improve the risk stratification of EC patients.

## Figures and Tables

**Table 1:** Distribution of sL1CAM levels according to clinicopathological characteristics in endometrial cancer patients.

	N. of Patients (%)			
Variable	sL1CAM-High	sL1CAM-Low	tot	*p*-Value ^1^
Age at diagnosis (years)				**0.005**
≤66	13 (35)	24 (65)	37	
>66	24 (69)	11 (31)	35	
FIGO stage ^2^				0.675
I–II	12 (48)	13 (52)	25	
III–IV	25 (53)	22 (47)	47	
WHO grading				0.303
Grade 2	5 (38)	8 (62)	13	
Grade 3	32 (54)	27 (46)	59	
Histology				**<0.001**
Endometrioid	14 (34)	27 (66)	41	
Non-endometrioid ^3^	23 (74)	8 (26)	31	
Myometrial invasion				0.535
M1	10 (56)	8 (44)	18	
M2	24 (47)	27 (53)	51	
Unknown	3	0	3	
Lymph node status				0.363
Negative	16 (52)	15 (48)	31	
Positive	12 (40)	18 (60)	30	
Unknown	9	2	11	
Lymphovascular invasion				0.284
Absent	3 (33)	6 (67)	9	
Present	31 (52)	29 (48)	60	
Unknown	3	0	3	
Risk Group				0.061
High–intermediate	3 (23)	10 (77)	13	
High	23 (55)	19 (45)	42	
Advanced Metastatic	11 (65)	6 (35)	17	

^1^ Significant *p*-values (*p* < 0.05) are shown in bold. ^2^ FIGO stage: 21 stage I, 4 stage II, 33 stage III, and 14 stage IV. ^3^ non-endometrioid EC: 15 serous, 5 clear cell, 10 mixed, and 1 undifferentiated.

**Table 2:** Uni and multivariate Cox regression analysis for disease-specific survival (DSS), progression-free survival (PFS), and platinum-free interval (PFI).

	DSS			PFS			PFI		
	HR	95% CI	*p*-Value	HR	95% CI	*p*-Value	HR	95% CI	*p*-Value
Univariate analysis									
Age (years)									
>66 vs. ≤66	2.14	1.06–4.32	**0.033**	1.88	1.02–3.46	**0.042**	1.71	0.93–3.16	0.083
FIGO stage									
III–IV vs. I–II	7.37	2.25–24.2	**0.001**	4.48	1.98–10.1	**<0.001**	4.44	1.96–10.1	**<0.001**
Histological Type									
Non-end vs. end	2.05	1.03–4.1	**0.042**	1.42	0.77–2.61	0.264	1.45	0.79–2.67	0.230
WHO grading									
G3 vs. G2	1.71	0.6–4.86	0.318	1.41	0.59–3.35	0.436	1.50	0.63–3.57	0.359
Myometrial invasion									
M2 vs. M1	1.89	0.72–4.94	0.193	1.70	0.78–3.70	0.132	1.79	0.82–3.91	0.141
Lymph node status									
Pos. vs. Neg.	3.51	1.39–8.85	**0.008**	2.08	1.02–4.27	**0.045**	2.16	1.05–4.42	**0.036**
LVSI ^1^									
Yes vs. No	5.78	0.79–42.4	0.085	1.62	0.58–4.57	0.358	1.69	0.60–4.75	0.321
sL1CAM									
High vs. Low	2.22	1.09–4.55	**0.028**	1.91	1.03–3.55	**0.041**	1.82	0.98–3.39	0.059
Multivariate analysis									
FIGO stage									
III–IV vs. I–II	8.46	2.56–27.9	**<0.001**	5.15	2.26–11.8	**<0.001**	5.22	2.28–12.0	**<0.001**
WHO grading									
G3 vs. G2	2.02	0.69–5.90	0.198	1.59	0.64–3.99	0.319	1.81	0.72–4.52	0.204
sL1CAM									
High vs. Low	2.13	1.03–4.40	**0.041**	1.93	1.01–3.70	**0.048**	1.75	0.91–3.37	0.095

^1^ LVSI: Lymphovascular space invasion. Significant *p*-values (*p* < 0.05) are shown in bold.

**Table 3:** Univariate and multivariate analysis of DSS, PFS, and PFI in EC patients stratified into three groups: sL1CAM-high endometrioid, sL1CAM-low endometrioid, and non-endometrioid tumors.

	DSS			PFS			PFI		
	HR	95% CI	*p*-Value	HR	95% CI	*p*-Value	HR	95% CI	*p*-Value
Univariate analysis									
EC groups									
sL1CAM-low EEC	1	-	-	1	-	-	1	-	-
sL1CAM-high EEC	2.90	1.02–8.18	**0.045**	2.58	1.08–6.16	**0.033**	2.69	1.12–6.47	**0.027**
non-EEC	2.81	1.25–6.32	**0.013**	1.83	0.92–3.63	0.086	1.89	0.95–3.76	0.069
Multivariate analysis									
EC groups									
sL1CAM-low EEC	1	-	-	1	-	-	1	-	-
sL1CAM-high EEC	2.96	1.03–8.50	**0.043**	2.45	1.02–5.88	**0.045**	2.97	1.22–7.21	**0.017**
non-EEC	3.17	1.36–7.38	**0.007**	2.06	1.01–4.22	**0.048**	2.31	1.13–4.72	**0.022**
FIGO stage									
I–II	1	-	-	1	-	-	1	-	-
III–IV	9.34	2.81–31.1	**<0.001**	5.36	2.33–12.3	**<0.001**	6.02	2.59–14.0	**<0.001**
Tumor grade									
G2	1	-	-	1	-	-	1	-	-
G3	2.09	0.71–6.17	0.182	1.73	0.70–4.24	0.233	2.02	0.83–4.93	0.124

EEC, endometrioid endometrial cancer; non-EEC, non-endometrioid endometrial cancer. Significant *p*-values (*p* < 0.05) are shown in bold.

## Data Availability

All data not included in this published article are available from the corresponding author upon reasonable request.
